# Biological Properties of Tocotrienols: Evidence in Human Studies

**DOI:** 10.3390/ijms17111682

**Published:** 2016-10-26

**Authors:** Puvaneswari Meganathan, Ju-Yen Fu

**Affiliations:** Nutrition Unit, Product Development and Advisory Services Division, Malaysian Palm Oil Board, 6 Persiaran Institusi, Bandar Baru Bangi, 43000 Kajang, Selangor, Malaysia; mpuvaneswari@gmail.com

**Keywords:** tocotrienols, human studies, clinical trial, biological properties, palm oil

## Abstract

Vitamin E has been recognized as an essential vitamin since their discovery in 1922. Although the functions of tocopherols are well established, tocotrienols have been the unsung heroes of vitamin E. Due to their structural differences, tocotrienols were reported to exert distinctive properties compared to tocopherols. While most vegetable oils contain higher amount of tocopherols, tocotrienols were found abundantly in palm oil. Nature has made palm vitamin E to contain up to 70% of total tocotrienols, among which alpha-, gamma- and delta-tocotrienols are the major constituents. Recent advancements have shown their biological properties in conferring protection against cancer, cardiovascular diseases, neurodegeneration, oxidative stress and immune regulation. Preclinical results of these physiological functions were translated into clinical trials gaining global attention. This review will discuss in detail the evidence in human studies to date in terms of efficacy, population, disease state and bioavailability. The review will serve as a platform to pave the future direction for tocotrienols in clinical settings.

## 1. Introduction

Due to the increasing trend of life expectancy and awareness towards lifestyle-related diseases, the nutraceutical industry is gaining prominence and has penetrated into consumers’ average daily diet. While the term nutraceutical has not been well defined, it generally refers to any food or supplements that have a beneficial nutritional effect. The current market trend has segmented the nutraceutical industry into two major categories, i.e., functional foods and dietary supplements. The global market size is estimated at USD 140.1 billion in 2010 [1]. Among the major global ingredients, vitamin E has market revenue of USD 83.4 million, alongside with omega-3 fatty acids, amino acids, probiotics and soy proteins [1]. While vitamin E is generally referred to α-tocopherol, the role of tocotrienols in human nutrition is frequently underestimated.

Vitamin E is a family of compounds consisting of two categories: tocopherols (TP) and tocotrienols (T3). Structurally, TP and T3 share a similar chromanol head. While tocopherols are attached with a saturated tail at the C2 position, tocotrienols have three double bonds in the side chain. Both TP and T3 have four homologs, namely alpha (α), beta (β), gamma (γ) and delta (δ). Nomenclature of the homologs is dependent on the degree and position of methylation at C5 and C7 position at the chromanol head. Although α-TP is widely known for its function in maintaining cardiovascular health, T3 tend to exhibit various health benefits beyond the antioxidant properties. Among the major sources of T3 are palm oil, annatto and rice bran oil [2].

**Figure 1:**
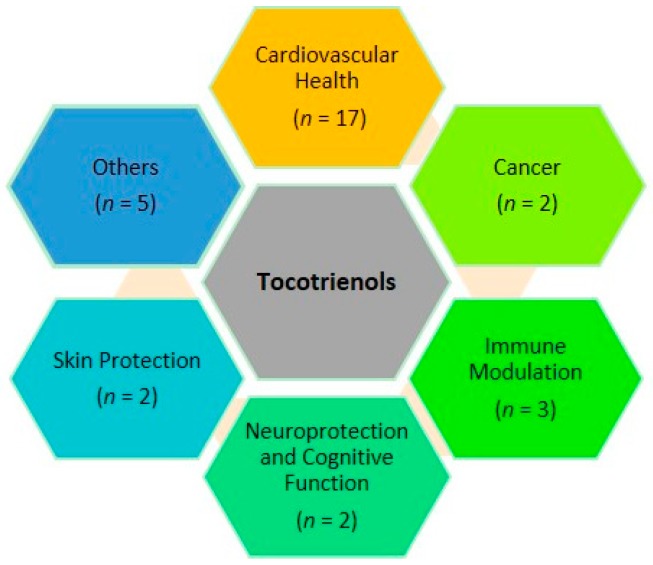
Therapeutic effect of tocotrienols studied/observed in clinical settings.

**Figure 2:**
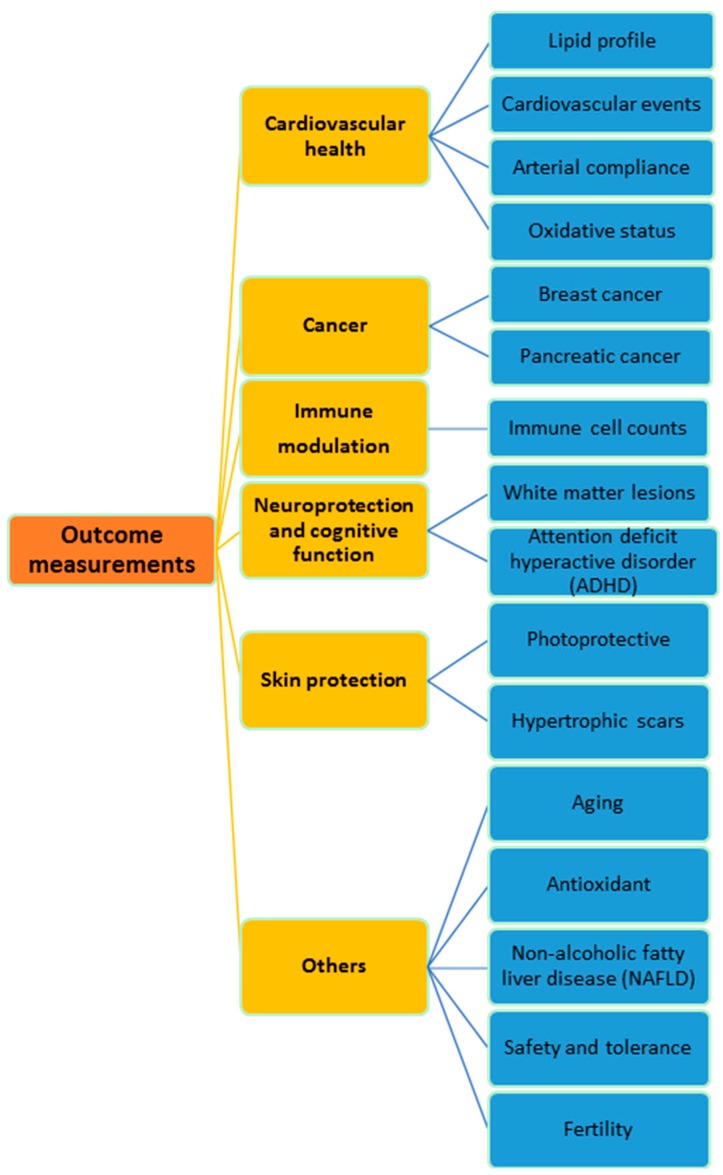
Summary of outcome measurements in tocotrienols clinical studies categorized based on their functionalities.

Global trend of nutraceutical ingredients is moving towards disease or condition specific formulations. Among the highly demanded formulations are those targeted for cardiovascular diseases, weight management, cognitive function, and bone/joint health. In this review, the clinical effects of T3 are summarized according to their functionalities. Supplementation of T3 in various population groups was found to show beneficial effects in cardiovascular health, cancer, immune modulation, neuroprotection and skin protection, as depicted in Figure 1 and Figure 2. Details of the clinical studies and research outcome will be discussed further in this review.

## 2. Cardiovascular Health

Association of reduced cardiovascular disease risk with long term vitamin E supplementation was well established among health practitioners despite contradictory findings on high dose α-TP supplementation [3]. While α-TP was found to reduce low density lipoprotein (LDL) oxidation primarily via anti-oxidant pathways [4], T3 were reported to exert their lipid-lowering effect beyond the anti-oxidant properties. *In vitro* study in liver cells and *in vivo* study in guinea pig showed inhibition of liver 3-hydroxy-3-methylglutaryl coenzyme A (HMG-CoA) reductase after T3 treatments, leading to regulation of cholesterol production [5,6]. Since then, a number of human studies was undertaken to determine the effect of T3 on cardiovascular health. Many of these studies investigated lipid profile as primary outcome, including total cholesterol (TC), LDL, high density lipoprotein (HDL) and triglycerides (TG). In trials involving hypercholesterolemic subjects, supplementation of tocotrienol-rich fractions (TRF) ranging from 200 to 300 mg per day resulted in significant decrease in TC and LDL [7,8,9,10,11]. Nevertheless, a dose dependent study of 60 to 240 mg of T3 per day was not able to demonstrate similar lipid lowering effect in 52 hypercholesterolemic subjects [12]. Another study involving 41 subjects of hypercholesterolemic men also did not find significant difference in the lipid profile after six weeks supplementation of TRF at 40 mg four times daily [13]. A possible explanation for the mixed results is insufficient dose that limits the blood concentration of T3 to exert an apparent effect. For example, when given in combination with lovastatin, a low dose of 50 mg TRF along with AHA Step-1 Diet was sufficient to lower the TC and LDL levels [9]. In this crossover study, combination of lovastatin and TRF showed additive effect in suppressing TC and LDL levels compared to either treatment alone. Besides, an observation reported by Qureshi et al. [7] found that the variability in response is likely to be attributed by inherent variation in serum cholesterol levels. Up to three non-respondents were identified out of the total 15 subjects in the study.

When studied in a different population, TRF seemed to have less lipid lowering effect in healthy subjects with normal cholesterol levels of TC < 6.2 mmol/L. Rasool et al. [14,15] and Chin et al. [16] reported the lack of significant changes in TC and LDL after supplementation of up to six months. However, Rasool et al. [14,15] demonstrated improved antioxidant status and arterial compliance upon supplementation with Tocotrienol-rich vitamin E (TRE). In the former study published by Rasool et al. [14], 80, 160 and 320 mg of TRE were supplemented to healthy volunteers. Meanwhile in the follow-up study [15], 50, 100 or 200 mg of self-emulsifying preparations of TRE were given to 36 healthy males for two months. Similarly, Chin et al. [16] reported a decrease in antioxidant enzymes including superoxide dismutase, catalase, and glutathione peroxidase following daily supplementation of 160 mg TRF for six months, especially in the age group above 50 years old. In a single arm study involving healthy subjects, significant reduction in TC was observed but no changes were detected for LDL and HDL after 30 days of supplementation with about 18, 42 and 240 mg of palm oil vitamin E concentrate (Palmvitee) [17]. Several clinical trials were also initiated to investigate the effect of T3 in diseased populations. In a pilot study where patients with type 2 diabetes mellitus were recruited and given 6 mg/kg/day of TRF, reductions in TC, LDL, and TG in addition to plasma glucose and glycated hemoglobin (HbA1c) levels were detected after 60 days [18]. While patients with end stage renal disease are associated with higher risk of cardiac events and accelerated atherosclerosis, a supplementation trial was conducted in 81 chronic hemodialysis patients [19]. Upon 180 mg/day of TRF supplementation for 16 weeks, significant reduction in plasma TG and apolipoprotein A1 were detected while changes in LDL and inflammatory markers were not apparent [19]. Among the few studies involving patients with previous history of cardiovascular events, Tomeo et al. [20] recruited a cohort of subjects with carotid artery atherosclerosis and supplemented the patients with 160–240 mg of TRF for 18 months. Disease progression determined using duplex carotid ultrasonography showed regression in the TRF group at 24% of patients by one category on the apparent degree of stenosis and 4% of patients by two categories, while 24% patients in the placebo group appeared to have exhibited progression of carotid atherosclerosis [20].

On the other hand, the four homologs of tocotrienols (α, β, γ, δ) were reported to have different potencies depending on the outcome measurement. For example, δ- and γ-T3 were more potent in cancer studies while α-T3 was more efficient in neuroprotection [21,22]. When translated into clinical trials, studies conducted using different T3 fractions showed distinctive results compared to TRF. Mustad et al. [23] conducted a study in hypercholesterolemic subjects supplemented with T3 containing high percentage of α- and γ-T3 with a dose equivalent to 200 mg per day. Results on the lipid profiles showed no difference in all lipid markers between intervention groups despite a marked increase in serum tocotrienol concentrations. In contrast, in a trial where subjects were supplemented with γ- and δ-T3, significant reduction in TG and very low density lipoprotein (VLDL) was observed although TC, LDL and HDL did not change much between groups [24]. In addition, when synthetically modified 250 mg/d of purified α-, γ-, or δ-tocotrienyl acetates was supplemented for eight weeks, only α-tocotrienyl acetate provided significant protection to LDL against oxidation. Conversely, the group supplemented with γ-tocotrienyl acetate showed increased levels of TC and LDL measured post-supplementation [25]. One of the limitations in studies using individual T3 fractions is the discrepancy in composition and purity. In most cases, the purity of the T3 fractions was not reported and the clinical effects from minor components such as desmethyl and didesmethyl T3 were conveniently neglected.

## 3. Cancer

Tocotrienols have gained the attention of clinicians by demonstrating compelling anti-cancer activities in pre-clinical research. The underlying mechanism facilitating this effect has been attributed to the anti-proliferative, anti-angiogenic, pro-apoptotic and immune enhancing nature of T3 [26]. The first clinical trial of tocotrienols in breast cancer patients was reported by Nesaretnam and colleagues [27]. In this five-year placebo controlled and double blinded study, 240 women with either Stage I or II estrogen receptor positive breast cancer were assigned to either placebo plus Tamoxifen or the treatment group which consisted of TRF with Tamoxifen. Women in the treatment arm took daily dose of 400 mg TRF. Upon completion of the study, there were six deaths and 20 local or systemic recurrence reported in the placebo arm while the TRF arm had only two deaths and 16 incidence of recurrence. Meanwhile, the five-year breast cancer specific survival was 98.3% in the TRF group versus 95% in the placebo group. Despite the mortality risk being lowered by 60% in the TRF arm, this finding was statistically insignificant due to the limitation encountered with small sample size being inadequate to detect breast cancer specific death between the two groups. All the women well-tolerated the TRF supplementation without any deviation reflected in their liver and blood parameters [27].

Consequently, in a recently published phase 1 dose escalation study, 25 pre-operative patients with pancreatic ductal neoplasia were given Vitamin E δ-Tocotrienol (VEDT) for two weeks prior to surgery [28]. The dose ranged from 200 to 3200 mg daily. Except for one patient with drug-related grade 1 diarrhea, the rest tolerated the treatment well without any dose limiting toxicity. The biological effective dose was assessed based on induction of apoptosis in neoplastic cells. Increased caspase-3 positive cells in the surgically removed tumor cells denote enhanced apoptotic activity and were associated with good biological effect response. The findings from this study showed that dose levels of 200 to 600 mg induced higher percentage of caspase-3 positive cells, which later reduced in the 800 to 3200 mg cohorts. The daily VEDT dose of 800 mg was chosen as statistically significant highest dose level with the biological effect response rate [28].

## 4. Immune Modulation

Modulation of the immune system has become an emerging field of interest in recent years due to its multi-targeted effect. One of the earliest clinical trial to study the immunomodulatory effect of T3 was conducted by Radhakrishnan et al. [29]. Healthy subjects supplemented with 200 mg TRF daily were assessed for levels of immune cells including T-helper, cytotoxic T-lymphocytes, CD4:CD8 ratio, B-lymphocytes and natural killer cells. Although the study failed to detect noticeable changes in the immune cells, a follow up study was designed to improve the outcome measurement. Mahalingam et al. [30] investigated the effect of TRF supplementation on immune response to tetanus toxoid vaccination in healthy volunteers. Subjects were given 400 mg TRF per day for two months and received an intramuscular injection of tetanus toxoid vaccine on day 28. The study found marked increment in anti-tetanus toxoid IgG in the TRF group. When challenged by tetanus toxoid, blood leukocytes from the TRF group showed significant higher production of interferon-gamma and interleukin-4 compared to placebo group. Although the effect of TRF might be specific to tetanus toxoid, this study served as an indication that T3 might be beneficial in enhancing immune response in healthy population upon immunogenic challenge. From a different perspective, Jubri et al. [31] investigated the role of TRF supplementation in the immune functions in cigarette smokers. Supplementation of TRF in smokers did not give rise to noticeable changes in total white cells, total lymphocytes, lymphocyte proliferation, and total T-cells, while an increase in the number of B-cells was observed in TRF supplemented non-smokers. Insufficient dose (200 mg per day) and raised basal values due to smoking were postulated to be the cause of unnoticeable changes in the outcome measurements [31]. Nevertheless, the study provided ample information on the distinctive patterns of immune functions between smokers and non-smokers in the Malaysian population.

## 5. Neuroprotection and Cognitive Function

Neuroprotective ability conferred by T3 in pre-clinical research has gained momentum and was further explored as a novel approach in humans. Beyond their antioxidative nature, T3, particularly α-T3, were found to induce neuroprotection at nanomolar concentrations. In fact, the circulating plasma T3 were found to be 20 times more than the required dose for neuroprotection [32]. Thereupon, Gopalan et al. [33] investigated the effect of TRF in white matter protection. Volunteers with white matter lesion (WML) were recruited and randomized into placebo or TRF groups. The volunteers in TRF group took 400 mg of TRF daily for two years and at the end of two years, the TRF group was reported to have statistically significant reduction in mean WML volume change as compared to the placebo group. This attenuation of WML and the safety of TRF as a supplement strengthened the neuroprotective ability of T3 and thus pave a path for future studies in larger population [33].

On the other hand, Attention Deficit/Hyperactive Disorder (ADHD) is a common behavioral disorder observed in school-going children. The medications commonly prescribed to reduce the symptoms are reported to cause adverse effects. Since oxidative stress has been identified as a known cause for neuronal damage that is modestly increased in ADHD, the efficacy of TRF, being a natural anti-oxidant, was investigated by Tan and colleagues in children with ADHD [34]. The children were supplemented with either 200 mg TRF or placebo daily for six months and were evaluated by validated questionnaires NICHQ Vanderbilt ADHD Parent Rating Scale (VAPRS) and NICHQ Vanderbilt ADHD Teacher Rating Scale (VATRS). The children were evaluated at baseline, three and six months. Although VAPRS showed improvement in both groups while VATRS showed improvement only in the TRF group, these results were not statistically significant. The limitation of this study might be attributed to supplementation at later age and the lack of an objective end point [34].

## 6. Skin Protection

Ultraviolet radiation is a common cause for oxidative damage of the skin by increasing the production of ROS which initializes a series of signaling cascade that aggravates the condition. Being an antioxidant, the photoprotective effect of T3 in combination with TP was investigated in photosensitive volunteers who were subsequently subjected to photoprovocative test in this trial [35]. Upon a single application prior to the provocative test, the topical formulation containing T3 was able to confer effective protection as compared to the vehicle and Vitamin A products. This finding has unveiled a new avenue for T3 as fewer studies have looked into the photoprotective nature of T3. Being a natural product, T3 is safe and well tolerated and formulations containing T3 could be investigated in more clinical trials in future. On the contrary, in another study, application of topical formulation consisting of T3 in post-surgical patients did not show any significant improvement on hypertrophic scar development after 6 weeks of application [36]. The differences in formulation, the composition of the active ingredients and severity of the condition might have caused these different observations. Nevertheless, no adverse event was reported during the study indicating that topical application of T3 was well tolerated [36]. Larger studies with variations in T3 formulations in variable degrees and types of skin diseases may answer the missing information in the topical protection conferred by T3.

## 7. Other Clinical Effects

In addition to the above functionalities, several exploratory studies found various response of TRF supplementation in different individuals. Heng and colleagues reported the effect of TRF supplementation and modulation of protein expression in young and old individuals [37]. Each group was further separated into placebo and TRF arm and supplemented for six months. In groups supplemented with TRF, blood levels of tocotrienols was increased in both the younger and older population with the younger group having higher concentration of plasma T3. An interesting finding from this study was despite having lower plasma T3 levels, the older group reflected greater changes in the number of proteins expressed. These proteins are mainly involved in cholesterol homeostasis (APOE and APOA1), acute-phase proteins (CRP), immune response (FHR1 and FHR2) and protease inhibitor (CBPN and AMBP). Increase in APOA1 and APOE proteins were associated with lowered risk of developing atherosclerosis and cardiovascular disease. Similarly, lowering of CRP also correlates with better prognosis for cardiovascular related diseases. Augmentation of immune response is one of the characteristics observed with TRF supplementation and, in this study, increase in FHR1 and FHR2 plasma proteins might be the underlying mechanism behind this beneficial effect.

In another study, the effect of TRF supplementation on DNA damage was investigated in individuals above and below 50 years old whereby the older group had higher DNA damage at baseline [38]. Supplementation of 160 mg of TRF daily for 6 months were reported to have markedly reduced DNA damage and increased chromosomal stability in older individuals as compared to those who are below 50 years old. Age related DNA damage that was profoundly elevated in the older individuals improved significantly upon T3 supplementation. The researchers postulated that, at cellular level, T3 may have either interfered in prevention of DNA damage or augmented DNA repair rate [38].

Increasing prevalence of Non Alcoholic Fatty Liver Disease (NAFLD) in both Asian and western nations led to initiation of a study to investigate the hepatoprotectivity of T3 in 64 individuals with NAFLD. After supplementation of 400 mg of TRF for a year, patients in the tocotrienol arm showed significant improvement in normalization of hepatic echogenic response in addition to absence of worsening of NAFLD grade [39]. On the other hand, two patients in the placebo arm were reported to show disease progression with worsening grade. Moreover, significant reduction of TC, LDL and TG levels was observed in the TRF arm compared to the baseline. All the patients tolerated the supplementation well without any adverse event [38]. Similar findings were reported in another study aimed at assessing the safety and tolerability of TRF in subjects with metabolic syndrome. Two weeks supplementation of 400 mg TRF daily did not cause any implication to hematological markers, serum liver function markers and liver enzymes. This implies that TRF supplementation does not cause hematoxicity and hepatotoxicity in metabolic syndrome subjects [40].

Mahdy and colleagues carried out a study to assess the association between early supplementation of TRF and the incidence of pregnancy induced hypertension (PIH) in healthy primigravidae recruited in their early second trimester and followed till delivery [41]. These women were supplemented with 100 mg TRF or placebo. The incidence of PIH although not significant but were found to be lower in TRF group with 64% reduction in the incidence of PIH. Remarkably, women in TRF arm were reported to have reduced blood loss in comparison with women on placebo. This finding dismisses the conventional concern that tocotrienols increases risk of bleeding and further asserts its safety [41].

## 8. Pharmacokinetics and Biodistribution

Despite promising findings on the health protective properties of T3, their bioavailability has been debated among health authorities. Several studies have been conducted to assess the fasting and postprandial levels of T3 in plasma. Yap and colleagues reported that food intake played a crucial role in the absorption of T3 [42]. The concentration of α-, γ- and δ-tocotrienols in plasma elevated significantly when taken with food in healthy individuals. A two-fold greater absorption was noted in the fed state based on the analysis of area under the curve (AUC_0–__∞_). Twice daily dosing was justified from the findings of this study as the half-life of tocotrienols was relatively much shorter than TP. Therefore, double dosing will ensure the presence of T3 in the circulating plasma for longer duration [42]. Another important factor to be considered in determining the bioavailability of T3 is the formulation which greatly influences the extent of absorption. The following study by the same team of researchers proved that self-emulsifying systems that produces finer droplets of emulsion prominently enhances the absorption of T3 by about two to three folds higher compared to non-emulsified formulation [43]. It was also observed that the lag time required for the secretion of bile salts to emulsify the T3 in order to facilitate their absorption were relatively shorter with the presence of self-emulsifying systems.

The findings by Yap et al. [42] were further confirmed by Fairus et al. [44], wherein the absorption of T3 was enhanced with food intake. Seven healthy subjects were administered a single-dose of either 1011 mg TRF or 1074 mg α-TP following a standardized breakfast. The major transporters of T3 were reported as triacylglycerol-rich particles (TRP) and HDL. However, the concentration of T3 was found to be much lower than TP in both circulating plasma and lipoproteins in this study. Likewise, comparable results were obtained in a follow-up study in 10 healthy volunteers supplemented with 526 mg TRF or 537 mg α-TP in similar study settings [45].

A study on the delivery and bioavailability of T3 to vital organs upon supplementation of 400 mg TRF ranging from one to 96 weeks in surgical patients was published by Patel and colleagues in 2012 [32]. Tocotrienol isoforms were found to be concentrated in circulating blood, skin, liver, cardiac muscle, brain and adipose tissue. Low bioavailability of T3 raised many uncertainties about their physiological role in humans. This study asserted that circulating levels of T3 were adequate and was higher than the therapeutic dose required to exert certain biological effects, such as neuroprotection. Another vital finding from this study was half of the individuals in the TRF arm reported reduction in model for end stage liver disease (MELD) score in comparison with only 20% in the TP arm [32]. In light of heightened risk of liver disease, the ability of T3 to delay the progression of the disease suggests a promising platform for future clinical trials to further ascertain the effects.

Most pharmacokinetic studies discussed above reported the concentration of individual isoforms upon supplementation with mixed T3 or TRF. In the following studies, newer formulations that consisted of T3 isoforms were investigated. These isoforms, namely gamma and delta tocotrienols were identified as the most potent compounds in pre-clinical anti-cancer research [46,47]. Springett et al. [28] studied (VEDT) in pancreatic cancer patients supplemented from 200 to 3200 mg daily for two weeks prior to surgery. The pharmacokinetic parameters such as Cmax, AUC_0–12_ and AUC_0__–∞_ increased upon VEDT supplementation from 200 to 1600 mg and declined at 3200 mg. Despite high level of dosage given, the subjects tolerated the supplementation well. Similar to Patel et al. [32] whereby oral supplementation of 400 mg/day of TRF reported to significantly increase the T3 concentration in blood and major organs as well as reduction in MELD score, evident physiological effect was observed with respect to VEDT supplementation indicating the favorable role exerted by tocotrienols. Increased apoptotic activity was reported with preoperative supplementation of VEDT in preoperative pancreatic cancer patients. In a different study, a formulation consisting of gamma and delta tocotrienols (GDT) were compared with conventional TRF in 12 healthy subjects in terms of bioavailability, safety and tolerability [48]. The gamma isoform in GDT showed superior bioavailability as compared to γ-T3 in TRF. All the volunteers tolerated the supplementation well indicating both the formulations were safe at given dosage.

In 2014, Fu et al. [49] reviewed the pharmacokinetics and bioavailability of T3 based on clinical studies conducted in the past two decades. Over the years, many issues that restrict and limit the absorption of T3 have been addressed and enhanced. Available evidence from human trials, suggests that T3 concentration in plasma increases upon supplementation and lower bioavailability is no longer a drawback factor for T3 to exert their therapeutic properties.

## 9. Conclusions

Based on this review, we understand that all clinical trials that had been conducted with T3 notably differ in their study designs, populations, formulations and dosing regimen. Despite the variations in the findings, these studies have facilitated our understanding on the potential physiological effect of T3. One of the major limitations of T3 debated in human trials was the lower bioavailability in plasma. Key findings from several studies have reported substantial concentration of T3 in plasma and vital organs, which are sufficient to exert health protective role in humans. Bleeding was a major concern with tocotrienols being labeled as anti-coagulant. Consistently, almost all the trials discussed have reported that T3 supplementation at different doses was safe and well-tolerated in the population studied. From healthy subjects to pre-surgical cancer patients and even pregnant women have tolerated T3 supplementation for short and long duration without any serious adverse event. With many of our queries and skepticisms on the bioavailability and physiological effect of T3 being answered, more studies are warranted to explore the therapeutic effect of T3 in various clinical settings and larger population to emphasize on the beneficial role of this natural product. The rising cost of drugs and the adverse effects commonly associated with them have led to many individuals and medical community to look for alternate solution. Tocotrienols being a well-tolerable natural compound with many distinct therapeutic properties may provide a possibility for better health care that may not only used to treat but may also play a major role in prevention. In order to materialize this idea into reality, more clinical trials are warranted to convey the convincing virtues of tocotrienols to the scientific community as well as the health care providers.

In line with the market trend, there is a shift of market demand towards natural ingredient and new delivery mechanisms [1]. Tocotrienols being a potent natural nutraceutical ingredient is a gift from nature. While more work on exploring new delivery systems will give added benefits to improve the bioavailability of tocotrienols, clinical evidence serve as the scientific fundamentals towards better acceptance among the consumers, clinicians and ultimately, the health authorities.
